# A novel report on the emerging and zoonotic neurotropic fungus *Trichosporon japonicum* in the brain tissue of the endangered Brazilian guitarfish (*Pseudobatos horkelii*) off the southeastern coast of Brazil

**DOI:** 10.1186/s12866-023-03128-w

**Published:** 2023-11-29

**Authors:** Maria Carolina Peixoto-Rodrigues, Gisela Lara da Costa, Tatiane Nobre Pinto, Daniel Adesse, Manoel Marques Evangelista Oliveira, Rachel Ann Hauser-Davis

**Affiliations:** 1 Laboratório de Avaliação e Promoção da Saúde Ambiental, IInstituto Oswaldo Cruz, Rio de Janeiro, Brazil; 2 Laboratório de Biologia Estrutural, Instituto Oswaldo Cruz, Rio de Janeiro, Brazil; 3 Laboratório de Taxonomia, Bioquímica e Bioprospecção de Fungos, Instituto Oswaldo Cruz, Rio de Janeiro, Brazil; 4 Department of Biochemistry and Molecular Biology, University of Miami Miller School of Medicine, Miami, FL USA

**Keywords:** Yeast, *Pseudobatos horkelii*, Elasmobranchs, *Trichisporon japonicum*, MALDI-TOF MS

## Abstract

Yeast infections have gained significant attention in the field of marine biology in recent years. Among the broad diversity of marine organisms affected by these infections, elasmobranchs (sharks and rays) have emerged as highly susceptible, due to climate change effects, such as increasing water temperatures and pollution, which can alter the composition and abundance of fungal communities. Additionally, injuries, or compromised immune systems resulting from pollution or disease may increase the likelihood of fungal infections in elasmobranchs. Studies are, however, still lacking for this taxonomic group. In this context, this study aimed to screen yeast species in cell cultures obtained from the brain of artisanally captured *Pseudobatos horkelii,* a cartilaginous fish that, although endangered, is highly captured and consumed worldwide. Fungi were isolated during an attempt to establish primary cultures of elasmobranch neural cells. Culture flasks were swabbed and investigated using morphological, phenotypic, and molecular techniques. Two isolates of the emerging opportunistic pathogen *Trichosporon japonicum* were identified, with high scores (1.80 and 1.85, respectively) by the MALDI-ToF technique. This is the first report of the basidiomycetous yeast *T. japonicum* in *Pseudobatos horkelii* in Brazil. This finding highlights the need for further research to determine the potential impact on elasmobranch health, ecology, as well as on commercial fisheries.

## Introduction

Significant shifts have increased in the marine environment due to climate change, leading in turn to salinity, light, temperature, sediment, and chemical pollution alterations. These changes result in marine fungi community imbalances, increasing the prevalence and severity of marine animal diseases due to fungal infections [1]. While over 10,000 pathogenic marine fungal species have been identified to date [2], and recently highlighted for their importance concerning marine health [3], their impacts in this regard remain largely understudied.

Certain fungi have been acknowledged as emerging opportunistic pathogens, such as *Trichosporon* spp. [4]. These organisms are widespread worldwide, with a preference for warm and tropical climates and are found in several environmental compartments, such as soil, decomposing wood and in both freshwater and marine environments, as well as in wildlife, such as bats, cattle and fish [5]. Members belonging to this genus have the potential to induce invasive and life-threatening fungal diseases in immunocompromised individuals [6, 7]. They also pose a risk to humans through the consumption of contaminated foodstuffs [8], including, but not limited, to milk and milk-derived products [9], honey [10], truffles [11] and seafood [12–17], as well as through workers exposed to potentially contaminated animals, such as zookeepers and fishers [18, 19].

Several aquatic biota, such as crustaceans and fish are highly vulnerable to fungal pathogens, both pathogenic and zoonotic [20]. Concerning *Trichosporon* spp. seafood contamination, assessments are mostly limited to crustaceans and bony fish, with reports on elasmobranchs, which encompass sharks and rays, however, still scarce.

Elasmobranchs are currently facing a multitude of threats, including overfishing, chemical pollution, habitat destruction, and climate change effects, resulting in global conservation concerns [21]. In this sense, the above-mentioned shifts in marine microbiota composition and diversity comprise a significant health effect factor that may negatively influence elasmobranch health and immune responses, potentially increasing their susceptibility to infections and diseases, increasing conservation concerns. In addition, many elasmobranch species are highly consumed as a protein source worldwide [22]. This also results in human health risks concerns, with potential human contamination by zoonotic pathogens posing as an important challenge, especially concerning specific vulnerable population strata, such as children and the elderly.

Although rays and skates are more threatened than previously estimated, with a higher percentage of species now being considered at risk compared to sharks [23], elasmobranch microbiome assessments have focused mainly on sharks to date. Furthermore, given their mostly benthic habits and direct association to substrates, many ray and skate species are at extremely high risk for contamination by several pollutants and pathogens, as sediments comprise the ultimate sink for these negative stressors [24–26].

In this context, brain samples from a highly endangered elasmobranch, *Pseudobatos horkelii*, commonly known as the Brazilian guitarfish, were collected as part of an ongoing project that aims to establish neural cell cultures from elasmobranchs. This species is distributed from Brazil to Uruguay, inhabiting coastal waters and occupying a meso-predator position, feeding on crustaceans, cephalopods, polychaetes and small fishes [27]. It is highly caught as bycatch and frequently consumed in Brazil and is categorized as Critically Endangered by the International Union for Conservation of Nature [28]. Although bans and extremely high fines have been implemented regarding this species capture and marketing in Brazil, significant numbers of these rays are sold for human consumption throughout the country (Hauser-Davis, Pers. Comm.).

Given that such cell cultures from this species were consistently contaminated by fungal species and dying at about 7 days post-plating, we decided to further investigate and describe which fungi may be present in the central nervous system of these elasmobranchs. The findings reported herein are highly relevant in describing potential emerging microbial pathogens that may directly affect elasmobranch health and may be transmitted to humans by elasmobranch meat ingestion and handling, also affecting primary studies requiring elasmobranch cell culturing.

## Methods

### Elasmobranch tissue isolation

*P. horkelii* specimens were captured by artisanal fishers in the state of Rio de Janeiro, southeastern Brazil. Only samples of recently deceased animals were sampled under a Brazilian Institute of Environment and Renewable Natural Resources Biodiversity Authorization and Information System (SISBIO) authorization, no. 77310–5.

Samples were obtained from two fresh specimens recently landed at an artisanal fisher colony in Rio de Janeiro, Brazil and displayed for sale on stainless steel display tables. Aiming to obtain sterile brain tissue, the animals’ heads were cleaned twice with 70% alcohol followed by cleaning with 2% benzalkonium chloride. Incisions were made on the animals’ heads with the aid of a #15 scalpel and sterile surgical scissors in the presence of a portable Bunsen burner and brain samples were collected. Tissue fragments of 10 by 10 mm^2^ were dissected from the telencephalon and placed in a transport medium, consisting of Leibovitz L-15 culture medium, 5% penicillin–streptomycin antibiotic solution, 0.25 ug mL^−1^ of fungizone, 50 ug mL^−1^ of gentamicin, 50 ug mL^−1^ neomycin sulfate, 373 mmol L-1 urea and 89 mmol L^−1^ NaCl (adapted from [29, 30]. Fragments were kept in glass Erlenmeyer flasks on ice throughout transportation to the Laboratory of Structural Biology at the Oswaldo Cruz Foundation.

At the laboratory, the samples were washed three times in a PBS solution modified for elasmobranchs containing 299 mmol L^−1^ urea and 68 mmol L^−1^ NaCl in a sterile penicillin–streptomycin antibiotic solution containing 0.25 ug mL^−1^ fungizone [30]. The tissue was then fragmented into small pieces of about 1 mm^3^ each and a 0.05% trypsin/0,02% Ethylenediaminetetraacetic acid (EDTA) solution was added for 10 min at 26 °C. After incubation, mechanical dissociation was performed by pipetting with a sterile glass Pasteur pipette and centrifugation to remove the trypsin solution. The obtained pellet was resuspended in culture medium and cell viability was determined by Trypan blue exclusion. All laboratorial procedures were performed in Biosafety level 2 cell culture hoods.

A total of 500,000 viable cells were then placed in 25 cm^2^ culture flasks and maintained with a culture medium adapted for elasmobranch cells according to [31], containing 50% Dulbecco’s Modified Eagle Medium (DMEM, ThermoFisher), 35% Leibovitz L-15, 15% Ham’s F-12 (ThermoFisher), 333 mmol L-1 urea, 188 mmol L-1 NaCl, 12% fetal bovine serum (Cultilab, São Paulo), 1% Glutamax, 2% penicillin–streptomycin antibiotic solution, 50 ug mL^−1^ gentamicin, 50 ug mL^−1^ neomycin sulfate, 0.25 ug mL-1 fungizone, 2 ng mL^−1^ Recombinant human epidermal growth factor (ThermoFisher) and 2 ng mL^−1^ Recombinant Human fibroblast growth factor (ThermoFisher) in an incubator at 26 °C. Occasionally, tissue fragments were also transferred to the cell culture flasks along with dissociated cells, and some adhered as explants in the flasks.

About one week after plating, cultures were consistently noted as contaminated. To identify which microorganisms were most predominant in elasmobranch neural cell cultures, swabs were sampled from the culture flasks after three days in vitro (3 div) and placed in 5 mL tubes containing a 0.9% saline solution. The samples were then taken to the Laboratory of Taxonomy, Biochemistry and Bioprospecting of Fungi in less than 30 min after collection and kept at 4 ºC until the moment of analysis.

### Microbiological sampling of elasmobranch cell culture-derived swabs

Samples were streaked onto Sabouraud Dextrose Agar (SDA) and incubated at 30 °C for 48 h for morphological assessments. All samples presenting growth on the SDA medium were then subcultured onto CHROMagar Candida (BD Difco) and colonies were interpreted according to the manufacturer’s instructions.

### Proteomic genotyping

In addition to morphologic and phenotypic tests, the obtained isolates were also identified by MALDI-TOF MS following Pinto et al. [32] and Oliveira et al. [33], employing α-cyano-4-hydroxycinnamic acid (CHCA, Fluka, Buchs, Switzerland) used as the matrix. Each sample was analyzed in triplicate from the same culture and from sub-cultures on alternate days. Samples were air-dried at room temperature prior to spectra acquisition. Spectra were obtained on a Microflex mass spectrometer (Bruker Daltonics, Bremen, Germany) using the Flexcontrol v. 3.0 software and spectra were imported and analyzed using the Maldi Biotyper v. 2.0 software (Bruker Daltonics, Bremen, Germany). The *Escherichia coli* DH5α strain was used for in situ protein extraction employed as the standard for MALDI-TOF MS external calibration, according to [34]. Results are expressed as log values ranging from 0 to 3. Logscore cutoff criteria were applied, where values of 1.7 are employed for reliable genus identification, and values between 1.8 and 2.0 are considered as confirmed species identification [35].

## Results And Discussion

**Fig. 1:**
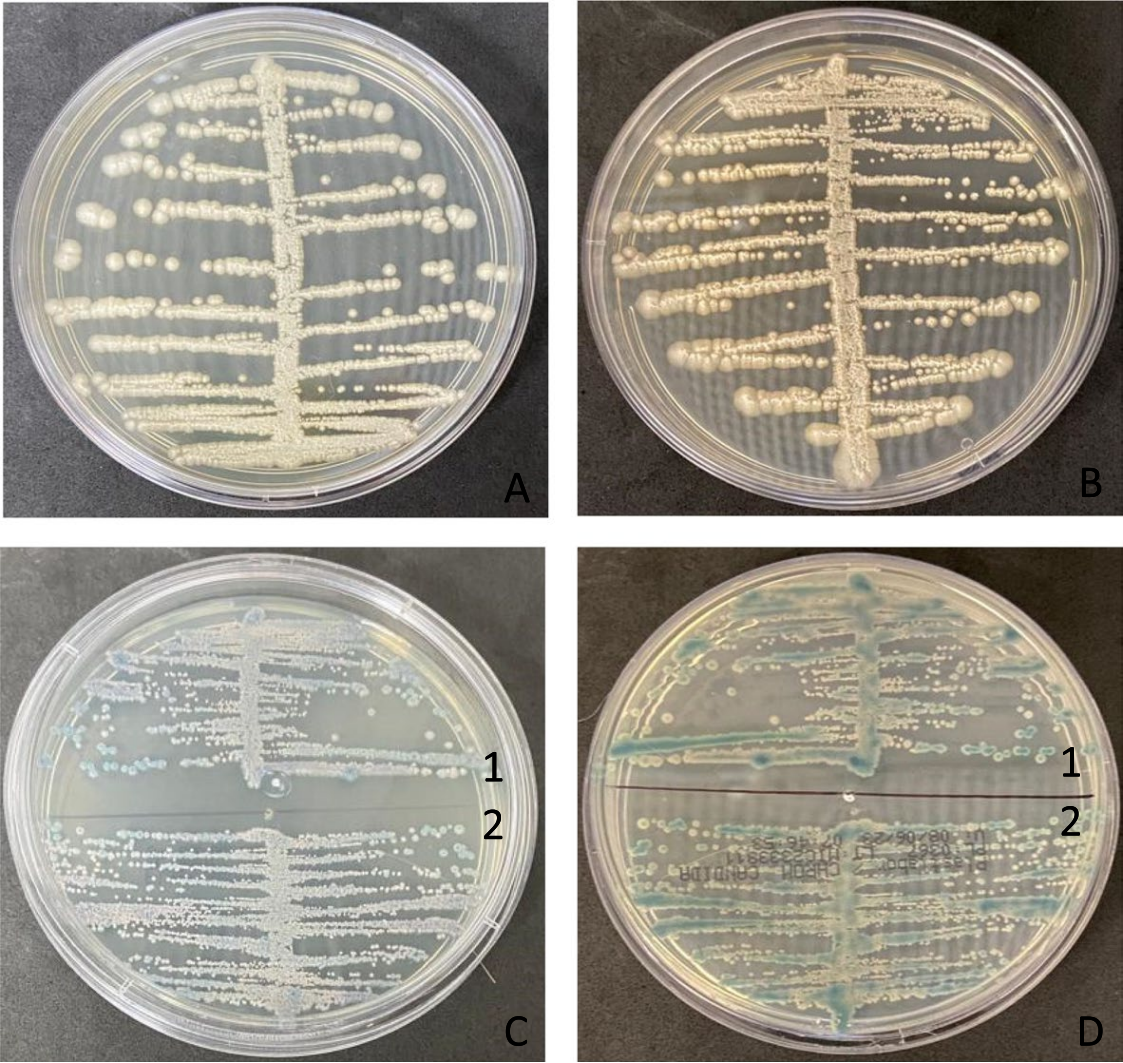
**A** and **B** Growths observed in Sabouraud Dextrose Agar incubated at 30o C for 48 h in aerobic conditions, samples LTBBF-RHO2 and LTBBF-RHO2; **C** and **D** Growths observed in BDTM CHROMagarTM Candida Medium (BD Difco) incubated at 35 oC for 48 h in aerobic conditions, front and back of the petri dishes, samples LTBBF-RHO2 (1) and LTBBF-RHO2(2)

The cell culture samples cultivated onto Sabouraud Dextrose Agar (SDA) as described above resulted in colonies with no bacterial characteristics detected in the SDA cultures. The SDA (Fig. 1A and B) growths were then further investigated by streaking onto CHROMagar *Candida* (BD Difco) plates. Small light blue-gray colonies (Fig. 1C and D), an atypical color for this substrate according to the manufacturer, were observed, indicating the growth of species other than *Candida* spp. Biochemical culture characteristics were then examined by conventional microscopy methods [32], and the morphological and phenotypical characteristics of the growths suggested the presence of *Trichosporon* sp.

Following microscopy identification, the samples were then analyzed by MALDI-ToF, which attributed high scores of 1.85 and 1.80 to the two isolate growths observed in the Sabouraud Dextrose Agar and in BDTM CHROMagarTM Candida Medium (BD Difco), identifying them at the species level as *T. japonicum*. MALDI-ToF analyses have been applied with high reliability to identify fungal species from several sources, including in a recent study by our group concerning another elasmobranch species also captured in Rio de Janeiro, Brazil, demonstrating high agreement with conventional identification methods and DNA sequencing [33]. Several studies have, in fact, noted that DNA sequencing is not required when a reliable spectral database is available [36, 37] and that the MALDI-ToF technique is, in fact, superior concerning yeast and mycobacteria isolate identification compared to gene sequencing. This, therefore, makes this technique suitable as a first-line test for yeast and mycobacteria identification [38]. This analytical technique is therefore a proven method for this type of fungal assessment in wildlife.

About 50 *Trichosporon* species are currently recognized, many of which are associated with human diseases [39]. *Trichosporon asahii* is the most commonly identified causative agent of trichosporonosis, a disease that exhibits both cutaneous and central nervous system-related manifestations [5], and other species, such as *T. cutaneum, T. asteroides, T. mucoides, T. inkin, T. jirovecii, T. dermatis, T. domesticum, T. montevideense, T. coremiiforme*, and *T. faecale* have also shown to be associated with infections [40]. Despite the use of antifungal therapy, the incidence of trichosporonosis in humans has increased over the past decades due to fungal resistance, leading to significant human morbidity and mortality rates ranging from 40 to 90% [5]. Some authors have postulated that this may be due to the increasing use of echinocandins, currently the drugs of choice in many clinical contexts at high-risk for invasive fungal infection [41].

The prevalence of fungal infections has, in fact, increased substantially worldwide, particularly among high-risk populations such as individuals with HIV/AIDS, transplant recipients, and those with weakened immune systems [42–44], although certain fungi, including *Cryptococcus*, *Coccidioides*, and *Histoplasma*, can also infect people with healthy immune systems [45–47]. For example, in the USA, *Candida* spp. caused 72 to 228 infections per million inhabitants annually, while *Cryptococcus neoformans* was responsible for 30–66 infections per million inhabitants, and *Aspergillus* spp., for around 12–34 infections per million inhabitants in the late 1990s and early 2000s [48]. More recently, the average occurrence of new *Candida* spp. infections was around 9 per 100,000 individuals between 2013 and 2017 and, according to the CDC, an estimated 25,000 cases of candidemia are reported annually throughout the United States [49]. The extent of central nervous system damage, however, varies based on the specific fungal forms present in the human body, such as blastopores or hyphae [44, 50, 51]. Fungi like Histoplasma, Blastomyces, Coccidioides, Candida, Paracoccidioides and Cryptococcus can enter capillaries and subarachnoid spaces, leading to conditions such as meningitis and subpial ischemic lesions. Candida, in contrast, has the potential to infiltrate blood vessels, causing localized necrotic lesions, while larger vessels may be breached by *Aspergillus*, Cladosporium, and Mucoromycetes, which could potentially result in strokes [52].

Conversely, the source of *Trichosporon* spp. infections remains uncertain, even though the incidence of infection by this pathogenic agent has increased globally in recent decades [5], especially due to increased immunosuppressive conditions and malignant hematological diseases [39, 53, 54]. *Trichosporon* spp. have been frequently detected in hospital environments, with a notable association between trichosporonosis and invasive clinical procedures, such as the use of probes and catheters [55]. For example, one study involving 17 patients revealed that 10 experienced fungemia with approximately 41% of these developing trichosporonosis after catheter usage [56]. Most suffered from acute leukemia and neutropenia, underwent chemotherapy and were receiving high doses of corticoids, which the authors indicate may have potentially influenced the development of trichosporonosis. Another study assessed the species distribution and antifungal susceptibilities of 22 bloodstream *Trichosporon* isolates recovered from patients hospitalized in five medical centers between 1995 and 2007 in São Paulo, Brazil [57]. The samples were obtained from both pediatric (44%) and adult patients presenting a diverse range of underlying conditions, such as premature birth, surgery, organ failures, inflammatory gastrointestinal disease, and cancer. Most patients (67%) developed systemic bacterial infections either before or concurrently with fungemia and had a central venous catheter in place when fungemia occurred. The authors indicate that these findings support the idea that invasive Trichosporon infections can occur in non-cancer patients with chronic illnesses and disruptions to skin and mucous membranes. The predominant species was *T. asahii*, followed by *T. larkias*, *T. coremiiforme*, *T. dermatis*, *T. inkin*, *T. ovoides*, and *T. mucoides*. Another investigation evaluated 24 clinical isolates of Trichosporon species isolated from blood, samples, pleural fluid and nails from 2005–2016 in a tertiary hospital in North India [58]. The isolates were identified as *T. asahii*, followed by *T. dermatis* (8.33%), *T. japonicum* (4.17%), *T. ovoides* (4.17%) and *T. mucoides* (4.17%). The authors indicated isolate resistance to fluconazole, voriconazole and itraconazole and susceptibility to ketoconazole, which is of concern due to the increasing number of cases of disseminated trichosporonosis being noted worldwide.

*Trichosporon* spp. are also, nevertheless, considered a normal inhabitant of the digestive tract and may be present on healthy patient skin [54, 59, 60]. In newborns, natural protective barriers, such as the skin and digestive mucosa, may become more permeable, potentially facilitating exposure to various pathogens, such as *Trichosporon* spp. In fact, *Trichosporon* spp. have already been detected on the skin of premature infants [61]. The mechanisms through which fungi can invade the central nervous system (CNS), though, have not yet been fully elucidated [62]. The blood brain barrier (BBB) is a critical component of the mammalian brain, formed primarily by the brain capillary endothelium under the influence of neighboring astrocytic glia [63]. It establishes a specialized microenvironment for optimal neuronal function and plays a crucial role in preventing harmful substances from entering the brain. Infection by *C. albicans* causes BBB disruption that can lead to transient encephalitis and cognitive impairment [64, 65], probably by crossing endothelial cells through the paracellular pathway while also being internalized by cells before exiting the basal surface through the transcellular pathway [66, 67]. *Cryptococcus* spp., in turn, employs several mechanisms to break through the BBB and access the CNS, also through facilitated paracellular passage. In vitro experiments, for example, have demonstrated that phagocytes containing viable *C. neoformans* can cross a layer of brain endothelial cells, indicating that *Cryptococcus* spp. can employ a “Trojan horse” strategy to infiltrate the brain [68].

Despite the fact that elasmobranch brains possess several similarities to mammalian brains [69], they differ concerning the BBB. While the endothelium of elasmobranchs does not act as a barrier to macromolecule entry, a glial BBB is suggested to exist [70]. Studies involving skates, dogfish, sharks, and rays have demonstrated that dyes are prevented from entering the brain but can pass through the endothelial layer [71]. These differences concerning mammalian and elasmobranch BBB probably interfere with brain permeability and may comprise an important factor in the invasiveness of pathogens like fungi, and in the establishment of CNS infections.

Although considered an exceedingly rare representative within the *Trichosporon* genus, clinical cases of contamination by *T. japonicum* have been reported in several regions, including Japan, where this species was initially discovered in 1971 from the atmosphere of a microbiological laboratory, as well as other countries [72–74]. The first documented clinical case of *T. japonicum* contamination occurred in 2008, involving a child diagnosed with acute myeloid leukemia (AML) who experienced fungemia associated with *T. japonicum* [75]. In Brazil, only two *T. japonicum* isolates have been isolated from humans [76] and one from artisanal cheese [77], making this only the third report of this fungal pathogen in the country, and the first in wildlife consumed by humans.

Fungal pathogens cause significant economic losses to aquaculture activities, increasingly posing risks to both cultured and wild fish populations worldwide [78], as farmed fisheries often drain their residues into rivers [79], and subsequently, the oceans. This may, therefore, potentially lead to local and pan-continental extinctions [80], presenting broader implications for global health, biodiversity, and conservation [81, 82]. This indicates the need for continuous monitoring of both freshwater and marine fungal fauna associations. Specifically concerning *Trichosporon* spp. fish contamination, one study reported the presence of *Trichosporon beigelii*, a novel fungal pathogen, in the cuticle of the freshwater crayfish *Astacus astacus*, raising concerns that crayfish may serve as a vector for this pathogen, significantly [83], while another study isolated *T. jirovecii* form the exoskeleton as well as eyestalks, gills, muscle and haemolymph of red swamp crayfish (*Procambarus larkia*) from the River Nile [16]. In another study, *T. mucoides* was isolated from the gills and intestine of Tilapia (*Oreochromis* sp., 4 positive isolates out of 27, 5.40%), the gills and intestine of African catfish (*Clarias gariepinus*, 3 positive isolates out of 22, 3.89%) and from the gills of gray mullet (*Mugil cephalus*, 2 positive isolates out of 21, 3.12%) [17]. Interestingly, fish feed has also been noted as contaminated with *Trichosporon asahii* [84], comprising another concern for aquaculture activities in this regard. As, however, bans are in place concerning *P. horkeili* marketing and consumption in Brazil, this report may also comprise an indirect conservation tool, as increased awareness of the potential contamination of these fish with zoonotic fungi may lead to reduced consumption of the critically endangered *Pseudobatos horkeili* in Brazil.

People routinely exposed to fungus-contaminated animals are at heightened risk for contracting fungal infections. One report, for example, indicated mycobacteriosis due to a finger wound caused by the dorsal fin spines of a fish (*Tilapia sp.*) netted during fishing activities by a fisher, which the authors postulated as being caused by either *Mycobacterium marinuum* or *Sporothrix shenckii* [85]. Other studies have reported the presence of several pathogenic fungi in the traumatogenic structures of freshwater fish, such as stings, rayed fins and teeth, and high incidence of fungal conditions in artisanal fishers [86], indicating occupation exposure concerns. As elasmobranchs are manipulated by fishers with no gloves, and usually sold decharacterized worldwide, *i.e.,* with no caudal fin or head, potential fisher fungal exposure is increased, causing further concerns regarding the detection of the rare *Trichosporon japonicum* in the *P. horkeili* specimens analyzed herein. Finally, this report may also comprise an indirect conservation tool, as increased awareness of the potential contamination of these fish with zoonotic fungi may lead to reduced consumption of the critically endangered *Pseudobatos horkeili* in Brazil.

## Conclusion

The method applied herein comprises an adequate tool to identify atypical and emerging fungal species in wildlife that may not be recognizable by conventional methods which is especially valuable in emerging fungal infections or cases where traditional methods might fail. The detected *Pseudobatos horkelii* contamination by *Trichosporon japonicum* indicate the possible ubiquitous contamination of Brazilian coastal waters by this rare fungus, suggesting that *Pseudobatos horkelii* may be an interesting sentinel species for emerging fungal pathogens in coastal marine waters. Furthermore, potential consumer and fisher contaminations may also occur. The limited understanding of elasmobranch associations with fungal pathogens remains and the significant lack of research on fungal biodiversity, prevalence, and their physiological impacts is concerning given the crucial role fungi may play in addressing climate change effects, the vulnerability of elasmobranchs, and the potential zoonotic nature of various fungal species. This clearly indicates the need for further assessments on the fungal diversity of elasmobranchs. This report also served as an indirect conservation tool by raising awareness about potential zoonotic fungi contamination, potentially reducing consumption of the critically endangered *Pseudobatos horkeili* in Brazil.

## Data Availability

The datasets used and/or analyzed during the current study are available from the corresponding author on reasonable request.
